# Time to regain birth weight - a marker to predict the severity of retinopathy of prematurity?

**DOI:** 10.1186/s12887-021-03027-x

**Published:** 2021-12-02

**Authors:** Ajay Anvekar, Sam Athikarisamy, Shripada Rao, Andy Gill, Elizabeth Nathan, Dorota Doherty, Geoffrey Lam

**Affiliations:** 1 Department of Neonatal Paediatrics, Fiona Stanley hospital, Perth, Australia; 2 Department of Neonatal Paediatrics, Perth Children’s Hospital, Perth, Australia; 3 Department of Neonatal Paediatrics, King Edward Memorial Hospital for Women, Perth, Australia; 4 Department of Paediatrics, University of Western Australia, Perth, Australia; 5 Women and Infants Research Foundation, King Edward Memorial Hospital for Women, Perth, Australia; 6 Division of Obstetrics and Gynaecology, University of Western Australia, Perth, Australia; 7 Department of Ophthalmology, Perth Children’s Hospital, Perth, Australia; 8 Centre for Ophthalmology and Visual Science, University of Western Australia, Perth, Australia

**Keywords:** Preterm, Birth weight, Retinopathy of prematurity

## Abstract

**Background:**

Poor weight gain in the first few weeks of life has been studied as a predictor of retinopathy of prematurity (ROP). Our aim was to assess whether time taken to regain birthweight (BW) be used as an additional marker to identify infants with type 1 ROP.

**Methods:**

In this retrospective study, preterm infants (< 27 weeks gestational age at birth) born during the period from 1/1/2010–31/12/2015 at a tertiary neonatal intensive care unit in Australia were included. Twenty-seven preterm infants with Type 1 ROP were identified. Controls (No ROP or ROP other than type 1) were matched with cases on gestational age at birth and BW (1:4 ratio). Data were collected from the database and medical records.

**Results:**

The median (IQR) gestational age for Type 1 ROP and control groups were 24 (24–26) and 25 (24–26) weeks respectively and median (IQR) BW for Type 1 ROP and control groups were 675 (635–810) and 773 (666–884) grams respectively. Preterm infants with Type 1 ROP were more likely to be small for gestational age (SGA) (18.5% vs 3.7%, *p* = 0.015) and had increased weeks on oxygen therapy (median 11.9 vs 9.1, *p* = 0.028). Time to regain BW was longer in preterm infants with type 1 ROP than controls but did not reach statistical significance (median 9 vs 7 days, OR 1.08, 95% CI 1.00–1.17, *p* = 0.059) adjusted for SGA and duration of oxygen therapy. The area under the curve from the time to regain BW model with adjustment for SGA and duration of oxygen therapy was 0.73 (95% CI 0.62–0.83).

**Conclusion:**

We hypothesize that time to regain BW has potential to aid prediction of Type 1 ROP and this warrants further investigation in a larger prospective study.

## Background

Retinopathy of prematurity (ROP) is a retinal neovascular disease seen in the low birthweight preterm infants [1]. Severe forms of ROP can lead to retinal detachment and blindness if not identified and treated at the right time [2]. ROP is one of the major causes of childhood blindness, in both developed and developing countries [3]. Improved survival of preterm infants and perinatal services has led to an increased incidence of severe ROP [3].

The aetiopathogenesis is complex and multifactorial. Arrest of the normal retinal vascular development is followed by an abnormal compensatory vascularization which leads to ROP. In phase 1 of ROP development, hyperoxia and loss of growth factors provided in utero to the fetus result in suppression of growth, and arrest of retinal vascularization. Subsequently, in phase 2, hypoxia causes stimulation of growth factor induced vasoproliferation in the increasing metabolically active, poorly vascularized retina [4].

These two phases result from alteration in levels of insulin like growth factor (IGF)-1 and vascular endothelial growth factor (VEGF) [5]. IGF-1 is needed for normal retinal vascularization [6–9] and it also influences VEGF- induced retinal vascular growth [7, 10]. Low IGF-1 following preterm birth due to loss from maternal sources and poor endogenous production [6, 11–13] impedes retinal vessel development. IGF-1 is regulated by calorie and protein intake [14]. Endogenous production of IGF-1 increases with age and size allowing VEGF activity, and proliferative retinopathy develops [15].

Prenatal factors such as premature placental dysfunction increase the risk of ROP in preterm infants following postnatal exposure to risk factors [16]. Low gestational age (GA) [17] and birthweight (BW) [18] are major risk factors for ROP. Excessive supplemental oxygen, sepsis, necrotizing enterocolitis, intraventricular hemorrhage, anemia, apnea, and blood transfusion have been described as risk factors for ROP and have been hypothesized to act by lowering serum IGF-1 levels [15].

The association between low IGF- 1 and poor postnatal weight gain in the development of ROP has been shown by the research work of Smith, Hellstorm et al. which have led to the use of postnatal growth as a surrogate measure for serum IGF-1 [19, 20]. Algorithms based on postnatal weight gain (WINROP, CHOP ROP, ROP score) have shown variable sensitivity in detecting infants at risk of developing ROP in different populations. These algorithms have the potential to reduce the number of eye examinations for ROP [15].

The risk of developing ROP has been shown to be associated with poor weight gain in the first few weeks of life. Extreme preterm infants are vulnerable to have low energy intake during their early neonatal intensive course, which has been associated with poor weight gain in the first 4 weeks of life [21]. Suboptimal postnatal weight gain in the first 2 weeks of life has been found to be an independent risk factor for ROP needing treatment.

Screening infants for ROP involves repeated ophthalmologic examinations by trained ophthalmologists, which are not free from adverse event [1]. Pain, stress to infants, hypoxemic and apneic episodes and feed intolerance have been associated with ophthalmic examinations for ROP [22]. Of all the infants undergoing screening, only 10% needed treatment for ROP [23]. Our study aimed to determine whether time taken to regain BW can be used as an early additional marker for identifying infants at risk of developing Type 1 ROP.

## Materials and methods

### Design and setting

A retrospective case control study in a tertiary level NICU in Australia

### Ethics approval

This study was approved by the hospital Governance Committee and the institutional research screening committee.

### Participants

**Fig. 1:**
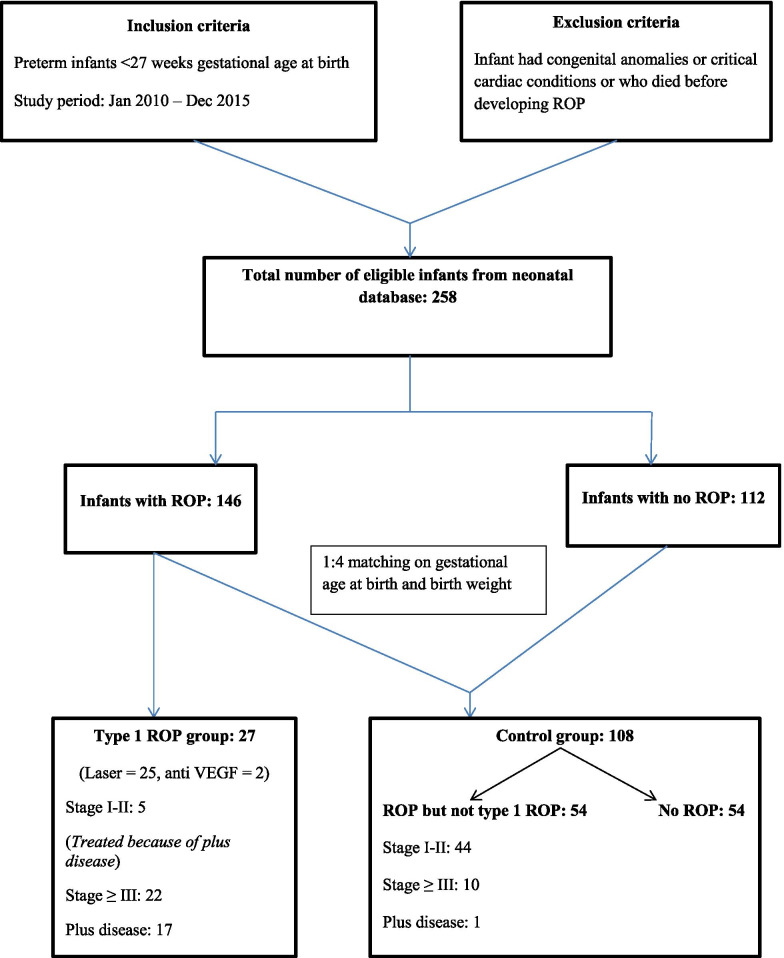
Study design

All preterm infants (< 27 weeks gestational age at birth) admitted between January 2010 and December 2015, were enrolled for analysis. Preterm infants who developed Type 1 ROP and needed treatment were identified. Controls defined as preterm infants who had No ROP or had ROP other than type 1 and hence no treatment. Controls were matched with cases on GA at birth and BW on a 1:4 ratio with equal distribution between no ROP and ROP but not type 1 (Fig. 1). Infants who had congenital anomalies or critical cardiac conditions and who died before developing ROP were excluded from the study.

### Data collection

The following data was collected from each infant’s medical record and neonatal database using a standardized, pre-piloted data collection form.

Antenatal risk factors: Chorioamnionitis, preeclampsia, diabetes mellitus, oligohydramnios, premature rupture of membrane and antenatal glucocorticoids.

Delivery details: Place of birth, GA, BW, gender, growth status, mode of delivery and Apgar scores.

Post-delivery care: Time to regain BW, duration of mechanical ventilation and noninvasive ventilation, duration of oxygen therapy, treatment for patent ductus arteriosus, necrotizing enterocolitis, postnatal steroids, need for blood transfusion, culture positive sepsis, duration of hospital stay, presence of intraventricular hemorrhage and periventricular leukomalacia on head scans and death before discharge were collected.

### Outcomes

The primary outcome was to determine whether the ‘time taken to regain BW’ can be a useful marker in identifying infants who developed Type 1 ROP. The secondary outcomes were to assess the effect of chorioamnionitis, blood transfusion, antenatal steroids, duration of oxygen therapy and culture positive sepsis as risk factors for Type 1 ROP.

### Guidelines for management of ROP in the NICU

#### Screening criteria

Neonates born < 31 weeks postmenstrual age, regardless of BW and neonates with BW < 1250 g, regardless of postmenstrual age at birth were screened for ROP. Neonates born greater than 31 weeks gestation at birth, BW > 1250 g, with additional problems, were screened at the discretion of the treating Neonatologist.

The screening examination for ROP followed the guidelines proposed by American Academy of Pediatrics Section on Ophthalmology; American Academy of Ophthalmology; American Association for Pediatric Ophthalmology and Strabismus and American Association of Certified Orthoptists [24] with amendments from local experience. The first screening examination was performed at 30 to 31 week postmenstrual age in infants born at < 27 weeks GA and at 4-5 weeks postnatal age in infants born at 27- to 31-week GA. After the first evaluation, if the subject did not have ROP, the neonate was evaluated at 2–4 week intervals until full vascularization. If the patient had active or rapid progressive lesion, the subject was evaluated more frequently, depending on the clinical findings.

Treatment for ROP in the unit was primarily laser surgery and the treatment decisions were based on the Early Treatment ROP guidelines. Laser was performed for retinal findings of Type 1 ROP (Zone 1 ROP: any stage with plus disease; Zone I ROP: stage 3, no plus disease; and Zone II: stage 2 or 3 with plus disease). Anti VEGF treatment was used for infants who were unwell for laser or if the disease process was posterior and laser was a risk for damaging ocular structures.

### Statistical method

Continuous data were summarized with medians, interquartile ranges (IQR) and ranges (R), and categorical data as frequency distributions. Conditional logistic regression analysis was used to univariately compare characteristics between cases and controls, and multivariable modelling to examine the effect of time to regain BW on Type 1 ROP. An assessment of potential confounders including chorioamnionitis, duration of oxygen therapy, blood transfusion, antenatal steroids and culture positive sepsis was made using a forward selection method to avoid over-parameterization. Univariate and adjusted effects were summarized as odds ratios (OR) and adjusted OR (aOR) respectively, with 95% confidence intervals (CI). The predictive potential of time to regain BW for Type 1 ROP was further assessed using area under the curve (AUC) constructed from probabilities generated from the conditional logistic regression models. Time to event data, including duration of continuous positive airway pressure, mechanical ventilation, nasal high flow and oxygen were summarized using Kaplan-Meier survival estimates and compared using the log rank test, with deaths before discharge censored for analysis. IBM SPSS (version 22.0, Armonk, NY) and Cytel Studio (version 8.0, 2007) statistical software were used for data analysis. *p*-values < 0.05 were considered statistically significant.

## Results

Twenty seven preterm infants with type 1 ROP [Laser (*n* = 25), Anti VEGF (*n* = 2)] were matched on a 1:4 ratio with 108 controls on GA and BW (Fig. 1). The median (IQR) GA for Type 1 ROP and control groups were 24 (24–26) and 25 (24–26) weeks respectively, and the median (IQR) BW for Type 1 ROP and control groups were 675 (635–810) and 773 (666–884) grams respectively. Preterm infants with type 1 ROP were more likely to be small for gestational age (SGA) (18.5% vs 3.7%, *p* = 0.015) and had increased weeks of oxygen therapy (median 11.9 vs median 9.1, *p* = 0.028). Other demographic and clinical characteristics were similar between the two groups. All in the type 1 ROP group (*n* = 27) and 103/108 (95.4%) controls were given blood transfusions (Table 1). Preterm infants with type 1 ROP had advanced stages of ROP compared to controls (Fig. 1).

**Table 1:** Demographic and clinical characteristics

	Type 1 ROP (***n*** = 27)	Control (***n*** = 108)	***P***-value
Male	19 (70.4%)	61 (56.5%)	0.274
Inborn	26 (96.3%)	103 (95.4%)	1.000
SGA	5 (18.5%)	4 (3.7%)	0.015
Chorioamnionitis	8 (29.6%)	20 (18.5%)	0.176
Antenatal steroids (complete)	18 (66.7%)	73 (68.9%)	0.927
Caesarean section	18 (66.7%)	49 (45.8%)	0.061
Apgar< 7 at 1 min	25 (92.6%)	90 (83.3%)	0.366
Apgar< 7 at 5 min	9 (33.3%)	30 (27.8%)	0.725
Time to regain birthweight	9 (6–13)	7 (5–10)	0.114
PDA treated	20 (74.1%)	79 (73.1%)	1.000
NEC	1 (3.7%)	6 (5.6%)	1.000
IVH	3 (12.0%)	4 (3.8%)	0.280
PVL	1 (12.5%)	1 (2.7%)	0.720
Culture positive sepsis	9 (33.3%)	28 (25.9%)	0.439
Mechanical ventilation (days)	30.8 (8.5–44.6)	11.3 (2.2–34.4)	0.191
CPAP (days)	44.6 (37.2–55.8)	44.8 (35.5–55.7)	0.997
Nasal high flow (days)	10.0 (0–19.3)	11.1 (0–18.8)	0.632
Duration of oxygen (weeks)	11.9 (7.0–17.9)	9.1 (4.0–13.4)	0.028
Postnatal steroids	2 (7.4%)	7 (6.5%)	1.000
Blood transfusion	27 (100%)	103 (95.4%)	0.622
Oxygen at 36 weeks	19 (70.4%)	64 (59.3%)	0.417
Home oxygen	3 (11.1%)	8 (7.4%)	0.763
Died during NICU stay	–	2 (1.9%)	1.000

Data represents median (IQR) or number (%). *IUGR* Intrauterine growth restriction, *CPAP* Continuous positive airways pressure, *PDA* Patent ductus arteriosus, *NEC* Necrotizing enterocolitis, *IVH* Intraventricular hemorrhage, *PVL* Periventricular leukomalacia, *NICU* Neonatal intensive care unit

**Fig. 2:**
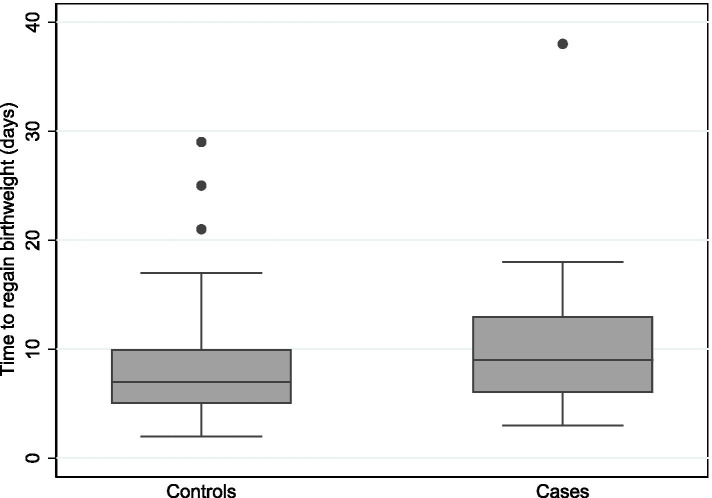
Box plot comparing time to regain birthweight on Type 1 ROP

**Fig. 3:**
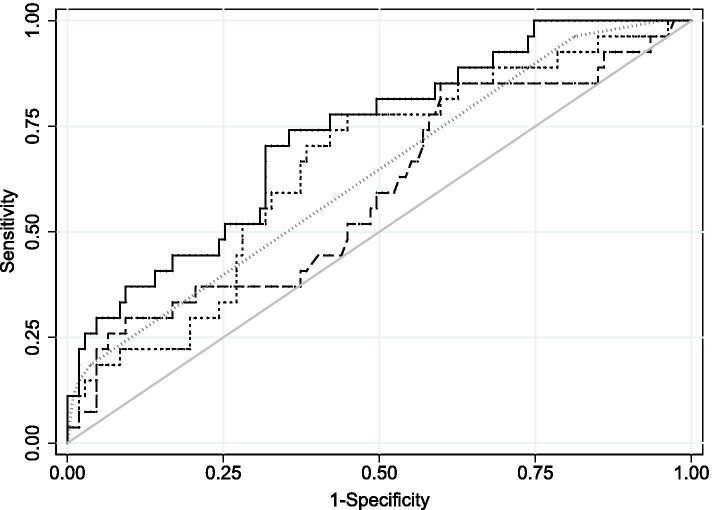
ROC curve illustrating predictive potential of time to regain birthweight, duration of oxygen therapy and SGA for developing Type 1 ROP

Median (IQR) time to regain BW in Type 1 ROP group and controls were 9(6–13) and 7(5–10) days respectively (Table 1, Fig. 2). On univariate analysis, there was no significant association between time to regain BW and the development of Type 1 ROP (OR 1.06, 95% CI 0.99–1.14, *p* = 0.108). The association of time to regain BW for developing type 1 ROP after adjusting for duration of oxygen therapy and SGA did not reach statistical significance (aOR 1.08, 95% CI 1.00–1.17, *p* = 0.059) (Table 2). Other risk factors assessed in the model were not statistically significant (all *p*-values> 0.05, Table 2). The AUC’s for the individual model covariates were: time to regain BW AUC: 0.60, 95% CI 0.47–0.72, weeks of oxygen therapy AUC: 0.65, 95% CI 0.54–0.77 and SGA AUC: 0.63, 95% CI 0.55–0.72. When time to regain BW was modelled simultaneously with oxygen duration and SGA, the AUC was increased to 0.73, 95% CI 0.62–0.83 demonstrating some predictive potential for Type 1 ROP (Fig. 3).

**Table 2:** Unadjusted and adjusted odds ratios (OR) and 95% confidence intervals (CI) for characteristics assessed in the multivariable model

	Unadjusted OR (95% CI)	Adjusted OR^a^ (95% CI)	***P***-value
Time to regain birthweight	1.06 (0.99–1.14)	1.08 (1.00–1.17)	0.059
Oxygen (weeks)	1.08 (1.01–1.17)	1.07 (0.98–1.15)	0.121
Chorioamnionitis	2.12 (0.72–6.25)	2.44 (0.73–8.12)	0.147
Antenatal steroids (complete)	0.96 (0.39–2.34)	1.06 (0.37–3.03)	0.916
Positive blood culture for sepsis	1.44 (0.57–3.60)	1.66 (0.61–4.54	0.322
SGA	14.44 (1.63–129.02)	9.29 (1.05–91.83)	0.055

^a^Adjusted odds ratios represent the covariate effect when fitted in the final model that included time to regain birthweight, duration of oxygen therapy and SGA

## Discussion

Our analysis of this retrospective data showed that the time to regain BW was longer in infants who needed treatment for ROP but didn’t reach statistical significance. However, AUC from our multivariable model for time to regain BW suggest that it may have the capability to be an additional marker for detecting Type 1 ROP. The possible reason for not reaching significance could be the low prevalence of ROP and small sample size. The infants in our study were extreme preterm (≤ 27 weeks PMA at birth) and had very low BW (≤ 1260 g) with preterm infants with type 1 ROP being more SGA than controls.

IGF-1, a peptide and its importance in prenatal, postnatal growth and role in development of retinal vasculature has been studied extensively. Postnatal growth being a good surrogate measure of serum IGF-1 has led to development of surveillance models which use GA, BW and postnatal weight gain to identify infants at risk of developing ROP. These models are limited by their sensitivity, lack of generalizability especially in non-Caucasian populations [25].

Association between postnatal weight gain and severe ROP has been studied by multiple investigators and it is important to discuss these studies. Earlier studies looked at the postnatal growth in the first four to 6 weeks of life and its association with severe ROP. A prospective study of 345 preterm infants by Filho et al. [26] (GA < 32 weeks, BW < 1500 g) found that poor weight gain at 6 weeks of life was a significant risk for ROP (small and appropriate for gestational age groups) and in a different study, Filho et al. [20] concluded that low weight gain by 6 weeks of age was a risk for severe ROP (*n* = 317). Further support for poor weight gain by 6 weeks of age and risk for severe ROP are supported by studies of Wallace et al. [27], Allegaert et al. [28] and Cabanas Poy [29]. Poor postnatal weight gain in the first 4 weeks of life was significantly associated with ROP needing treatment in a retrospective study of 233 preterm infants. Infants gaining 10 g/day had a risk reduction of 2.76–8.34% vs 7.17–12.76% in infants gaining 20 g/day [30]. Association of poor weight gain in the first 4 weeks with severe ROP is further supported by studies of Li et al. [31] and Saric et al. [32]. Studies from developing nations also have shown that decreased weight gain proportions at 4th and 6th weeks of life are risk factors for severe ROP [33, 34].

Four studies have looked at the relationship between postnatal weight gain in the first 2 weeks of life and ROP. In a prospective study by Aydemir et al. [35], the infants with severe ROP had significantly lower weight gain at 2 and 4 weeks postnatal age (*P* = 0.041 and *P* = 0.017 respectively) and time to regain BW was longer in infants with severe ROP (*P* = 0.003). Culture proven sepsis, blood transfusion volume, days on mechanical ventilation and oxygen supplementation were significant risk factors in the severe ROP group [35]. Kim et al. [3] compared early postnatal weight gain in association with ROP needing treatment (48 preterm infants with ROP requiring treatment vs. 163 preterm infants with no ROP or ROP requiring no treatment). On univariate analysis, time to regain BW, blood transfusions and oxygen supplement days were significantly higher in the ROP needing treatment group. They also noted that relative weight gain in the second, fourth and sixth week of life was significantly lower in the group needing treatment for ROP. On logistic regression, duration of oxygen supplementation (FiO2 > 0.4) and relative weight gain in second week of life were the only significant risk factors [3]. A retrospective study by Wongnophirun et al. found that infants with severe ROP needing treatment had lower relative weight gain and total calorie intake at 2 weeks of age [36]. Vinekar A et al. [25] looked at the time to regain BW in more mature preterm neonates (mean GA = 31 ± 2 weeks) in the NICU’s (*n* = 68) of a developing country. The authors found that number of days to regain BW was significantly different between no ROP vs. type 1 and no ROP vs. type 2 and type 1 vs. type 2 (*P* < 0.001). A risk stratification of their data (based on number of days taken to regain BW) showed that those who regained BW less than 10 days, 11–20 days and > 20 days had low, moderate and high risk for type 1 or 2 ROP respectively [24]. In our study, time to regain BW was longer in infants who developed type 1 ROP which is similar to observations noted in studies which have looked at early postnatal weight gain and ROP [3, 25, 35].

It is important to discuss growth status at birth. We had higher number of SGA infants in Type 1 ROP group compared to controls (18.5% vs. 3.7%, *p*-value: 0.015). Studies have found inconsistent results for SGA being a risk factor for severe ROP. Filho et al. [26] and Chu et al. [37] found SGA not to be a risk factor for severe ROP, while Allegaert et al. [28] reported SGA to be a risk factor for developing threshold ROP. However, a recent systematic review and meta-analysis found SGA to be associated with increased odds for severe ROP and suggested SGA to be considered in risk assessment of ROP evaluation [38].

The limitations of this study are its retrospective nature, small sample size, lack of data on weight gain when neonates where in critical condition and weight gain after the neonate reached BW. The limitations associated with low prevalence and small sample size also limit the ability to detect differences, especially with binary outcomes. Although our result is statistically inconclusive, we cannot rule out a true effect exists in the association between time to regain birthweight and ROP.

## Conclusion

Time to regain BW may have potential to increase the predictive ability for detecting Type 1 ROP. Phase 1 of the ROP pathogenesis corresponds to the time to regain BW. It is possible that the infants who developed Type 1 ROP were at a higher risk of low energy intake during the immediate to early postnatal period which possibly resulted in IGF-1 mediated vascularization arrest. We suggest that it is worthy to investigate our hypothesis in a larger prospective study.

## Data Availability

The data that support the findings of this study are available from the authors upon reasonable request.
